# Remote Monitoring in Myasthenia Gravis: Exploring Symptom Variability

**DOI:** 10.1002/acn3.70293

**Published:** 2025-12-21

**Authors:** Maike Stein, Maximilian Mönch, Meret Herdick, Frauke Stascheit, Sarah Hoffmann, Hannah Preßler, Philipp Mergenthaler, Carla Dusemund, Paolo Doksani, Haoqi Sun, Pushpa Narayanaswami, Andreas Meisel, Lea Gerischer, Sophie Lehnerer

**Affiliations:** ^1^ Department of Neurology With Experimental Neurology Charité—Universitätsmedizin Berlin, Corporate Member of Freie Universität Berlin and Humboldt‐Universität zu Berlin Berlin Germany; ^2^ Neuroscience Clinical Research Center Charité—Universitätsmedizin Berlin, Corporate Member of Freie Universität Berlin and Humboldt‐Universität zu Berlin Berlin Germany; ^3^ Berlin Institute of Health at Charité—Universitätsmedizin Berlin Berlin Germany; ^4^ Department of Neurology Beth Israel Deaconess Medical Center/Harvard Medical School Boston Massachusetts USA; ^5^ Institute of Biometry and Clinical Epidemiology Charité—Universitätsmedizin Berlin, Corporate Member of Freie Universität Berlin and Humboldt‐Universität zu Berlin Berlin Germany; ^6^ Center for Stroke Research Berlin, Charité—Universitätsmedizin Berlin Berlin Germany; ^7^ Radcliffe Department of Medicine University of Oxford Oxford UK

**Keywords:** disease progression, monitoring, myasthenia gravis, remote consultation, telemedicine

## Abstract

**Background:**

Myasthenia gravis (MG) is a rare, autoimmune disorder characterized by fluctuating muscle weakness and potential life‐threatening crises. While continuous specialized care is essential, access barriers often delay timely interventions. To address this, we developed MyaLink, a telemedical platform for MG patients. This study evaluated whether frequently assessed clinical outcomes via MyaLink can capture symptom variability between clinical visits.

**Methods:**

In this randomized, controlled, 12‐week study, 45 MG patients received either standard care (control, *N* = 15) or standard care with additional telemedical treatment (intervention, *N* = 30) including assessment of patient‐reported outcome measures, sensor‐based data, and patient‐physician messaging via a mobile application. Physicians performed telemedical check‐ups (TCUs) via a web‐based platform, adjusting treatment as needed.

**Results:**

In the intervention group, variability in clinical scores and sensor‐derived data was observed, particularly among those who experienced MG‐related hospitalizations or exacerbations (H&E subgroup). This subgroup showed larger MG‐ADL score fluctuations (mean range: 5.8 vs. 2.6 points), sent more messages, had more steroid dose adjustments (40% vs. 0%), and more frequent TCU interventions (93.3% vs. 60%) than the Non‐H&E subgroup.

**Interpretation:**

Telemedical platforms in MG might detect early signs of worsening. High‐risk patients (H&E subgroup) require increased medical support, which can be effectively addressed through MyaLink.

**Trial Registration:**

The study was registered under the German clinical trial registry (DRKS00029907)

**Abbreviations**
AChR: acetylcholine receptor
AES: advanced encryption standard
app: application
CFS: Chalder Fatigue Scale
CI: confidence interval
FcRn: neonatal Fc receptor
f.l.t.r.: from left to right
FVC: forced vital capacity
HADS: hospital anxiety and depression scale
IGQ+: intervention subgroup with additional QMG
IGQ−: intervention subgroup without additional QMG.
iMZ: integrated Myasthenia center
iOS: iPhone operating system
IQR: interquartile range
IVIg: intravenous immunoglobulins
LRP4: lipoprotein receptor‐related protein 4
MDD: Medical devices directive
MG: Myasthenia gravis
MG‐ADL: Myasthenia gravis activities of daily living
MGFA: Myasthenia gravis foundation of America
MGFA‐PIS: MGFA post‐intervention status
MG‐QoL15r: Myasthenia gravis Quality of Life, revised version
MuSK: muscle specific kinase
PASS: patient acceptable symptom state
PROM: patient‐reported outcome measure
QMG: quantitative Myasthenia gravis score
REDCap: research electronic data capture
SD: standard deviation
SSQ: single simple question
TCU: telemedical check‐up
vs.: versus

## 1 Introduction

Myasthenia gravis (MG) is a rare, chronic neurological disorder characterized by specific autoantibodies targeting the post‐synaptic membrane of the neuromuscular junction [1]. Clinically, MG manifests as exercise‐dependent muscle fatigability potentially leading to life‐threatening myasthenic crises [2]. Due to its chronic nature, the majority of MG patients require long‐term and often lifelong highly specialized care. Studies have shown a significant burden of disease [3, 4, 5] and a high demand for individual counseling on various topics [6, 7]. Despite advances in treatment, the care landscape for MG remains fragmented and complex. Access to MG specialists is often limited due to geographical constraints [8], and complicated as patients have to navigate between multiple healthcare professionals. Currently, much information about symptoms is lost between in‐person appointments and across sector boundaries. This lack of coordination and specialist accessibility can result in delays in treatment, avoidable hospitalizations, and poor health outcomes, particularly for patients on new immunomodulatory therapies (e.g., FcRn inhibitors, complement inhibitors) that require frequent adjustments. 10%–15% of MG patients experience at least one crisis requiring mechanical ventilatory support during their life [9]. Some crises may be preventable through timely and appropriate treatment [10]. However, current monitoring practices rely on brief and infrequent in‐person assessments that are unlikely to occur at times of patient‐perceived need due to rigid appointment scheduling. Remote monitoring using smartphone and wearable technology to collect ongoing health data outside the traditional care setting and for physicians to be able to review data in real time offers a promising approach to bridge gaps in care by complementing clinical evaluations and providing personalized support [11]. Such technologies have shown positive impacts on patient safety and quality of life in other indications, including reduction in hospital admission/readmission, length of stay, and number of outpatient visits [12, 13], but have yet been largely underexplored in MG. Beyond single‐domain digital tools, several digital monitoring initiatives are currently being investigated in MG. ME&MGopen applies wearable sensors and a mobile application for continuous symptom assessment [14] while BioDigit‐MG integrates patient‐reported, physiological, and behavioral data to assess disease activity [15]. We developed a remote monitoring platform, MyaLink, specifically tailored to MG, comprising a web‐based portal for physicians and a mobile application (“app”) for patients.

The app enables individual monitoring through patient‐reported outcome measures (PROMs) and biologic data from external devices (digital spirometer, wearable device), automatically integrated in MyaLink. MyaLink differs from existing approaches through its integrated, real‐time physician dashboard and secure bidirectional communication channel, which enables telemedical intervention and management rather than solely passive data collection [16]. This is an analysis of the data from the clinical MyaLink study to assess how frequent PROMs and parameters from external devices can capture symptom variability between clinical visits and provide physicians with information in real time to change management as indicated.

## 2 Methods and Materials

### 2.1 Telemedicine Platform

The telemedicine platform used in this study, MyaLink, was developed by Charité Universitätsmedizin Berlin and Qurasoft GmbH (software partner). The patient organization German Myasthenia Association (Deutsche Myasthenie Gesellschaft) was involved in the whole development process and provided advice on the patients' perspective. MyaLink is a data protection‐compliant, certified medical product (Medical device directive, MDD I). The platform includes an app for patients (available for iOS and Android) and a web‐based platform for physicians as well as a chat module. Patients monitor their symptoms through the assessment of PROMs (as detailed in Section 2.5) alongside simultaneous integration of sensor‐based physiological data transmitted via Bluetooth from connected external devices. These devices include a digital spirometer (MIR Spirobank Smart One) for measuring forced vital capacity (FVC) and a wearable wristband activity tracker (Garmin Vivosmart 5) measuring step count, oxygen saturation and heart rate. Further modules of the app include data storage of documents and a medication plan with reminder function. The web‐based portal allows physicians access to all monitoring data in real‐time and facilitates direct communication with patients via the chat, enabling timely therapeutic adjustments and management of MG as necessary.

### 2.2 Standard Protocol Approvals, Registrations and Patient Consent

This study was approved by the ethics committee at Charité Universitätsmedizin Berlin (EA2/157/22). The study was conducted in accordance with the declaration of Helsinki. Patients provided written consent. The study was registered under the German clinical trial registry (DRKS00029907).

### 2.3 Eligibility Criteria

Inclusion criteria were age ≥ 18 years, a Myasthenia Gravis Foundation of America (MGFA) status classification I–IV [17] and a diagnosis of MG for at least six months, identified from a retrospective review of medical records at the integrated Myasthenia center (IMZ). Diagnosis of MG was based on clinical features of fatigable muscle weakness supported by at least one of the following: antibody positivity, typical electrophysiological testing (detection of decrement or increased jitter), and/or clear response to anticholinesterase inhibitors. Exclusion criteria included the presence of medical conditions that could interfere with the patient's ability to report MG symptoms or affect the patient's ability to use the MyaLink platform, particularly if such conditions were not medically stabilized or were associated with cognitive dysfunction.

### 2.4 Patient Recruitment and Randomization

45 patients were recruited from the iMZ at Charité Universitätsmedizin Berlin. Patients were randomized in a 2:1 ratio into the intervention group (*n* = 30) and the control group (*n* = 15) using a digital randomization tool [18]. Randomization was stratified by sex (gender was additionally assessed during the baseline visit and no discrepancies between recorded sex and gender were observed in any patient) and the MGFA status classification at the time of enrollment (combined as MGFA I‐II and MGFA III‐IV).

### 2.5 Study Design

The study design was outlined in detail in a previous publication [16]. The intervention group and control group were followed over a 12‐week observation period between April and September 2023. All patients underwent two comprehensive visits at the study center at baseline and end of study, including a regular clinical follow‐up visit. During these visits, detailed data on MG history, clinical symptoms, comorbidities, medication, MG‐related hospitalizations, self‐reported exacerbations (validated through the physician consultation during the study visits), and care‐related aspects in MG were collected. Patients completed various PROMs and underwent a neurological exam focused on MG, including a standardized physician‐reported outcome measure (Quantitative Myasthenia Gravis Score, QMG, 0–39 point scale) [19]. MGFA status classification was assessed during baseline visit [17] and both MGFA status classification and MGFA post‐intervention status (MGFA‐PIS) [17] were assessed during the end of the study visit. Patients in the intervention group had the option to undergo three additional QMG [19] assessments, spaced 3 weeks apart during the study. Based on their choice, they were further categorized into either the IGQ‐ subgroup (intervention group without additional QMG, *n* = 12) or the IGQ+ subgroup (intervention group with additional QMG, *n* = 18).

At baseline, patients of the intervention group (both IGQ+ and IGQ‐) downloaded the MyaLink app and received a wearable (wristband activity tracker) and a digital spirometer. PROMs and reminders for spirometry measurements were assigned through the app at predefined intervals: Weekly PROMs/measurements included MG‐ADL (Myasthenia gravis Activities of Daily Living, 0–24 point scale) [20], MG‐QoL15r (Myasthenia gravis Quality of Life‐15 item, revised, 0–30 point scale) [21], SSQ (Single Simple Question, 0%–100%) [22] and reminders for spirometry measurements (assessing FVC). Monthly PROMs included CFS (Chalder Fatigue Scale, 0–33 point scale) [23], HADS (Hospital anxiety and depression scale, 0–42 point scale) [24] and PASS (Patient Acceptable Symptom State, “yes” or “no” response) [25]. If patients were hospitalized due to MG, additional QMG assessments were performed as feasible. Two telemedical check‐ups (TCUs) were conducted at week four and eight after baseline, where physicians reviewed monitoring data via the web‐based portal and contacted patients as needed (e.g., change in medication). All data from the study visits and the TCUs were documented in REDCap software (Research Electronic Data Capture, Version 13.7.31).

### 2.6 Statistical Analysis

The primary endpoint of this study was feasibility (measured by adherence rates for digital PROMs assessment) and usability and has previously been published [26]. All further secondary endpoints analysis, including this analysis of longitudinal remote data caption and clinical endpoints, is exploratory. The following research questions will be evaluated in further analyses, but are not part of this analysis:

1. Digital biomarkers: Can telemonitoring and wearable data be valuable to predict impending crises and exacerbations in patients with MG?
2. Communication patterns and telemedical intervention analysis: What are communication patterns and topics discussed between patients and physicians in the MyaLink chat? What insights can be gained from telemedical interventions?
3. Remote assessment of respiratory function: Is the single breath count test a feasible tool for remote assessment of respiratory function in MG?

The statistical analyses of the herein presented results were performed using R software (version 4.2.2) and R Studio software (version 2023.03.1 build 446) [27]. Since this study is exploratory, the analyses are conducted using the full data set without any imputations. Descriptive statistics (mean and standard deviation (SD), median and interquartile range (IQR), absolute numbers and percentages) were applied to summarize patient characteristics and study protocol‐defined endpoints. These endpoints, as outlined in the DRKS registry, ethics application and study protocol, include MG‐related hospitalizations, self‐reported exacerbations and MG‐specific scores and questionnaires. MG‐related hospitalizations and exacerbations were defined a priori as secondary clinical outcomes in the study protocol. The subsequent comparison of patients with and without these events (“H&E” vs. “Non‐H&E” subgroup) was not pre‐specified and therefore represents an exploratory, descriptive post hoc analysis. Exacerbations were reported by patients at the end‐of‐study visit and retrospectively validated through a physician interview during that visit (i.e., systematically assessed the reported episode and symptoms). Only events consistent with an exacerbation—as interpreted by the physician—were included and documented. Additionally, MGFA‐PIS for both groups is compared descriptively via bar charts including absolute and relative frequencies. Longitudinal changes in clinical outcomes within the intervention group are presented in spaghetti charts, including median and IQR over time. Individual changes in clinical scores from baseline to end of study are compared descriptively between the intervention and control group using mirrored histograms for the MG‐specific outcomes. Group effects were assessed as Cohen's d, derived from linear mixed‐effects models with repeated measures (random intercept models, random intercepts for patients) adjusted for baseline values, time point of measurement, interaction of time point and group allocation. Due to the absence of normally distributed variables, the coherence of the results was checked by non‐parametric tests for repeated measures data in factorial design using the nparLD package in R [28]. Any significant results will be reported by additionally stating the *p*‐value, but non‐significant outcomes will omit the *p*‐value.

## 3 Results

### 3.1 Patient Characteristics

45 patients were enrolled (intervention group: *n* = 30, control group: *n* = 15). One patient had incomplete follow‐up assessments (drop out in the intervention group, due to non‐MG related reasons). Demographic characteristics such as age, gender, and disease duration were well balanced between both groups (Table 1). The majority of patients were female (73.3%), and the median age of all patients was 48.0 years (IQR 42.0, 58.0). Median disease duration was five years (IQR 2.0, 9.0). Patients in the intervention group were more severely affected according to MGFA status classification, with 16.7% classified as MGFA IVb at baseline in the intervention group compared to 6.7% in the control group.

**TABLE 1:** Patient characteristics.

	Overall *n* = 45	Intervention group *n* = 30	Control group *n* = 15
Age, in years			
Median [IQR; min–max]	48.0 [42.0, 58.0; 23–82]	47.5 [40.5, 57.5; 23–82]	48.0 [45.0, 56.5; 25–64]
Disease duration, in years			
Median [IQR; min–max]	5.0 [2.0, 9.0; 1–17]	5.5 [2.0, 9.8; 1–17]	5.0 [1.5, 8.0; 1–13]
Sex/Gender^a^, *n* (%)			
Female	33 (73.3)	21 (70.0)	12 (80.0)
Male	12 (26.7)	9 (30.0)	3 (20.0)
MGFA status classification, *n* (%)			
I	1 (2.2)	1 (3.3)	0 (0.0)
IIa	8 (17.8)	5 (16.7)	3 (20.0)
IIb	9 (20.0)	6 (20.0)	3 (20.0)
IIIa	6 (13.3)	3 (10.0)	3 (20.0)
IIIb	15 (33.3)	10 (33.3)	5 (33.3)
IVa	—	—	—
IVb	6 (13.3)	5 (16.7)	1 (6.7)
Thymectomy, *n* (%)	32 (71.1)	21 (70.0)	11 (73.3)
Thymoma, *n* (%)	4 (12.5)	2 (9.5)	2 (18.2)
Antibody status, *n* (%) (multiple answers possible)			
AChR	33 (73.3)	20 (66.7)	13 (86.7)
MuSK	1 (2.2)	1 (3.3)	0 (0.0)
LRP4	2 (4.4)	2 (6.7)	0 (0.0)
Seronegative	9 (20.0)	7 (23.3)	2 (13.3)
Multiple antibodies, *n* (%)			
Double‐seropositivity^b^	1 (2.2)	1 (3.3)	0 (0.0)
Comorbidities, *n* (%)	37 (82.2)	24 (80.0)	13 (86.7)
If yes, which type (multiple answers possible)			
Autoimmune	17 (46.0)	10 (40.0)	7 (50.0)
Pulmonary	10 (27.0)	7 (28.0)	3 (21.4)
Psychiatric	14 (37.8)	8 (32.0)	6 (42.9)
Neurological	17 (46.0)	12 (48.0)	5 (35.7)
Other comorbidities	19 (51.4)	16 (64.0)	3 (21.4)

Abbreviations: AChR, acetylcholine receptor; FVC, forced vital capacity; IQR, interquartile range; IVIg, intravenous immunoglobulin; LRP4, lipoprotein receptor‐related protein 4; MG‐ADL15, Myasthenia gravis activities of daily living; MG‐QoL15r, Myasthenia gravis Quality of Life‐15 item, revised version; MGFA, Myasthenia Gravis Foundation of America; MuSK, Muscle specific kinase; PLEX, plasma exchange; QMG, quantitative Myasthenia gravis score.

^a^ Both categories assessed during baseline, and no discrepancies between recorded sex and gender were observed in any patient.

^b^ This included one patient in the intervention group with AChR‐ and LRP4 antibody positivity.

### 3.2 No Intervention Effects Regarding Clinical Scores Between the Intervention and Control Group

**FIGURE 1:**
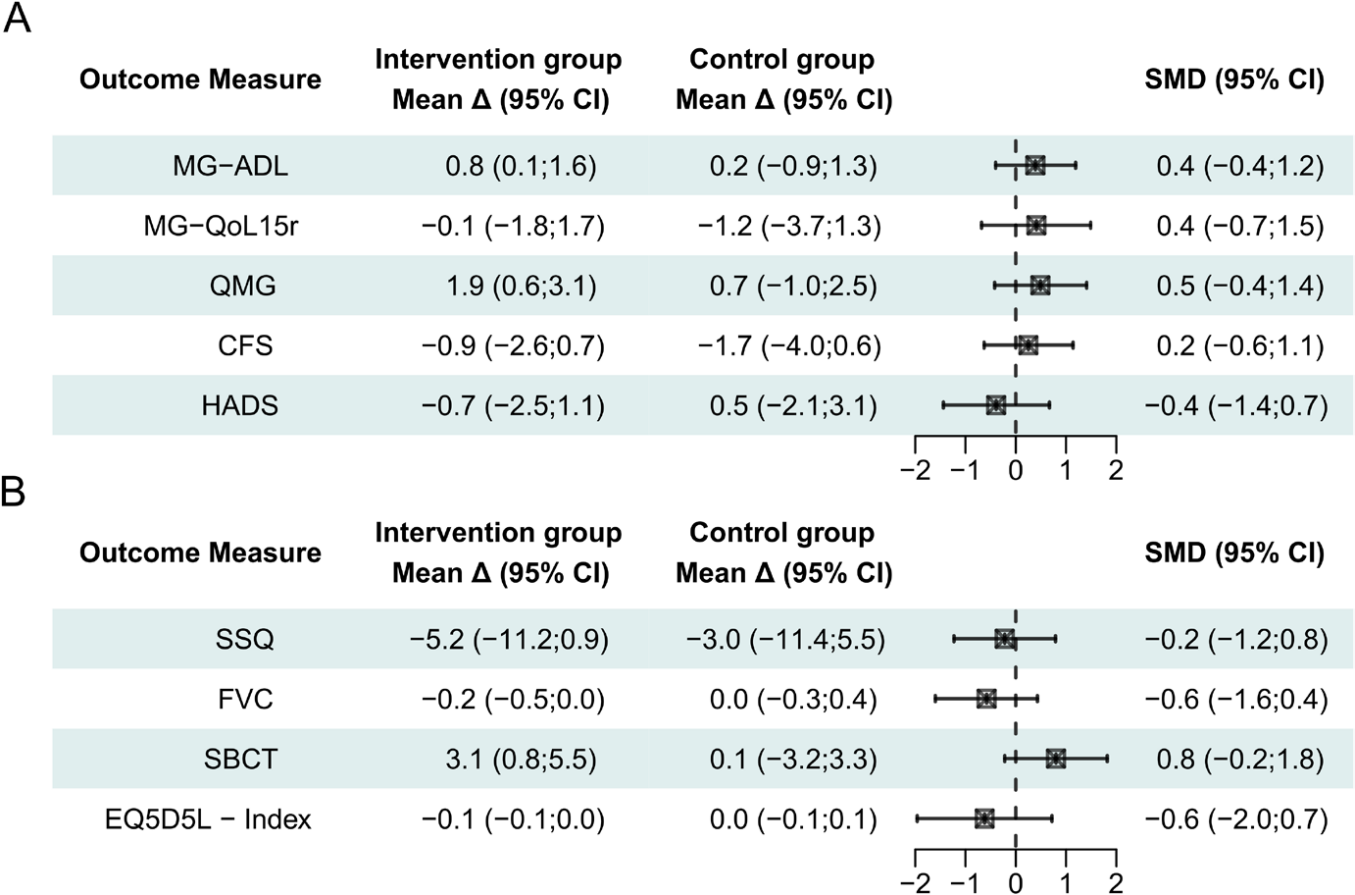
End of study group effects in clinical scores between intervention group and control group. Estimated mean change in scores (deltas Δ) at the end of study visit for each group and the resulting standardized mean differences (SMD) for treatment effect analyses (linear mixed‐effects models adjusted for baseline values, time point of measurement, and interaction of time point and group allocation). CFS, Chalder Fatigue Scale; EQ5D5L, health‐related quality of life, developed by the EuroQol Group; FVC, forced vital capacity; HADS, Hospital Anxiety and Depression Scale; MG‐ADL, Myasthenia gravis activities of daily living; MG‐QoL15r, Myasthenia gravis quality of life, revised version; QMG, quantitative Myasthenia gravis score; SBCT, single breath count test.

Comparison of the mean estimated adjusted clinical scores at the end of study demonstrated small intervention effects regarding all PROMs between the intervention group compared to the control group (Figure 1, raw data for clinical scores for both groups at baseline and end of study, as well as the within‐group differences between both visits are shown in Table S1). This demonstrated no statistically significant differences in any MG‐specific clinical outcome measures between both groups. MGFA‐PIS at the end of study was *unchanged* status in most patients (control group: *n* = 11, 73.3%; intervention group: *n* = 25, 86.2%), few patients worsened (control group: *n* = 2, 13.3%; intervention group: *n* = 4, 13.8%) and few patients improved (control group: *n* = 2, 13.3%; none in intervention group) (Figure S1). In both intervention and control groups, considerable variability in individual change of clinical scores from baseline to end of study for MG‐ADL, QMG, and MG‐QoL15r was observed, most pronounced on MG‐QoL15r (Figure S2).

### 3.3 MG‐Related Hospitalizations and Exacerbations in the Intervention and Control Group

15 patients (51.7%) in the intervention group had self‐reported exacerbations and/or MG‐related hospitalizations during the study (13 patients with exacerbations; five patients with hospitalizations excluding planned admissions, e.g., due to infusion therapies), compared to eight patients (53.3%) in the control group (eight patients with exacerbations; one patient with hospitalization excluding planned admissions, e.g., due to infusion therapies). Patients in the intervention group reported fewer exacerbations than in the control group (44.8% vs. 53.3%, OR = 0.72, CI: 0.17 to 2.96) but the intervention group had a higher rate of MG‐related hospitalizations compared to the control group (17.2% vs. 6.7%, OR = 2.9, CI: 0.3 to 27.6). In the intervention group, 62.5% of hospitalized patients had a stay > 14 days and received rescue therapy (IVIg and/or plasmapheresis/immunoadsorption) (Table S2).

### 3.4 Interindividual Variability Across Different Remote Monitoring Parameters in the Intervention Group

**FIGURE 2:**
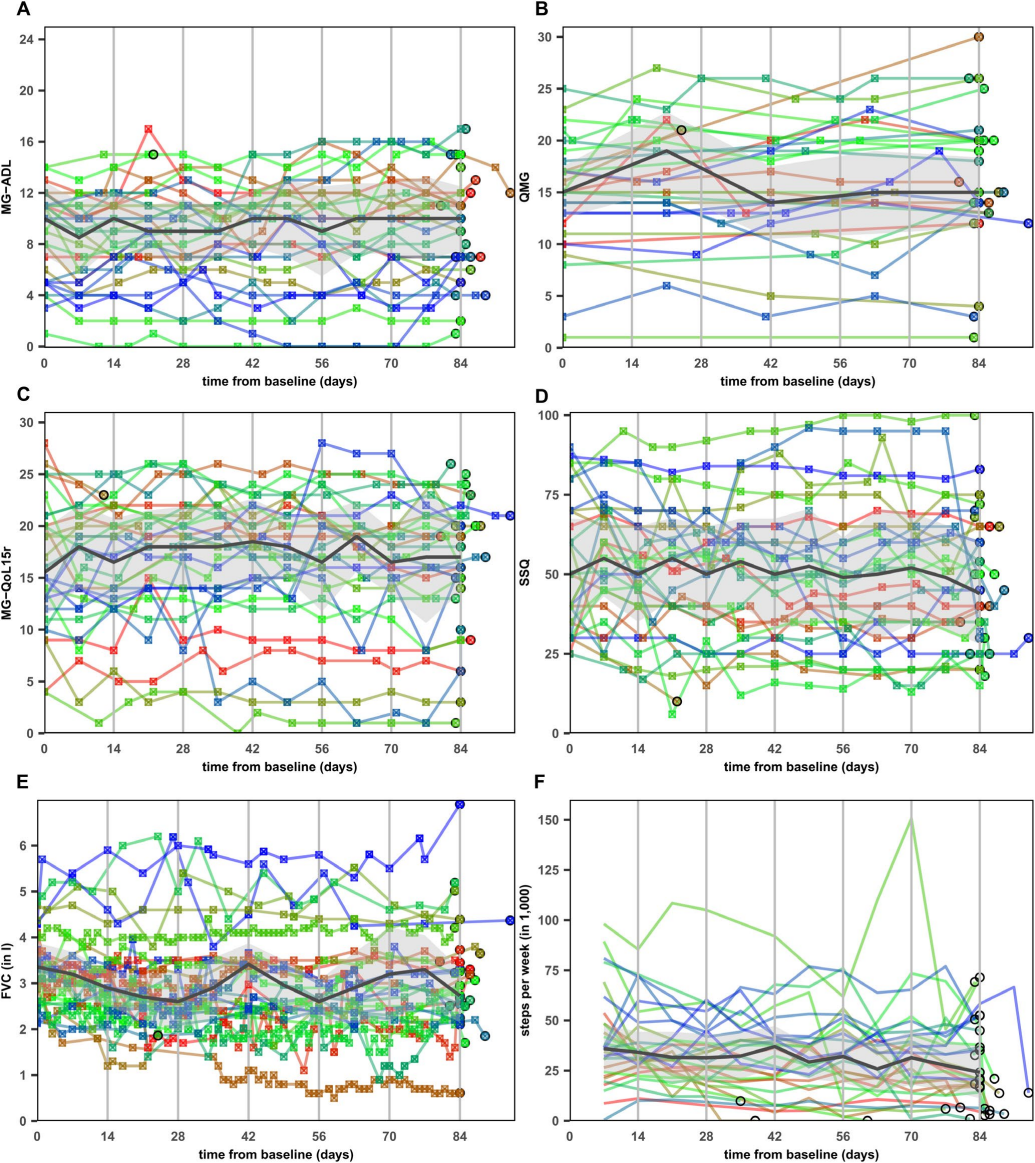
(A–F) Longitudinal changes in monitoring parameters. Longitudinal changes in outcome measures for each patient in the intervention group (*n* = 30). The last measurement per patient is marked with a circle and, except for steps, each timepoint of measurement is marked as a box. The black line enshrouded in a gray ribbon represents median values (black line) and IQR for each measurement. For the median of weekly measurements of MG‐ADL (Myasthenia gravis Activities of daily living), MG‐QoL15r (Myasthenia gravis Quality of Life, revised version), and SSQ (Single Simple Question), the values up until the scheduled measurement have been considered. For QMG (Quantitative Myasthenia gravis score), median values were determined using measurements within ± ten days of the scheduled measurement (every three weeks). For FVC (Forced vital capacity), only measurements at the scheduled day have been considered for the median. The measured steps have been summarized to cumulated steps per week for better visualization. No data imputation was performed.

Within the intervention group, longitudinal changes in PROM scores, spirometry (FVC), and wearable‐derived weekly step counts showed interindividual variability. Some patients maintained relatively stable scores over time, while others showed greater fluctuations (Figure 2A–F). Notably, interindividual variability was observed across all PROMs, particularly for MG‐QoL15r and SSQ. Many patients used the spirometer much more frequently than the required weekly assessment. Different patterns of interindividual monitoring parameters changes were also observed in the intervention subgroup of patients who experienced either MG‐related hospitalizations and/or self‐reported exacerbations (“H&E subgroup”, patients with none such events in the intervention group are called “Non‐H&E subgroup”) (Figure S3). Moreover, some patients (e.g., 34, 38) showed increased spirometer usage during hospitalization periods compared to non‐hospitalized periods.

### 3.5 Ranges of Clinical Score Variation in the H&E and Non‐H&E Subgroups

**FIGURE 3:**
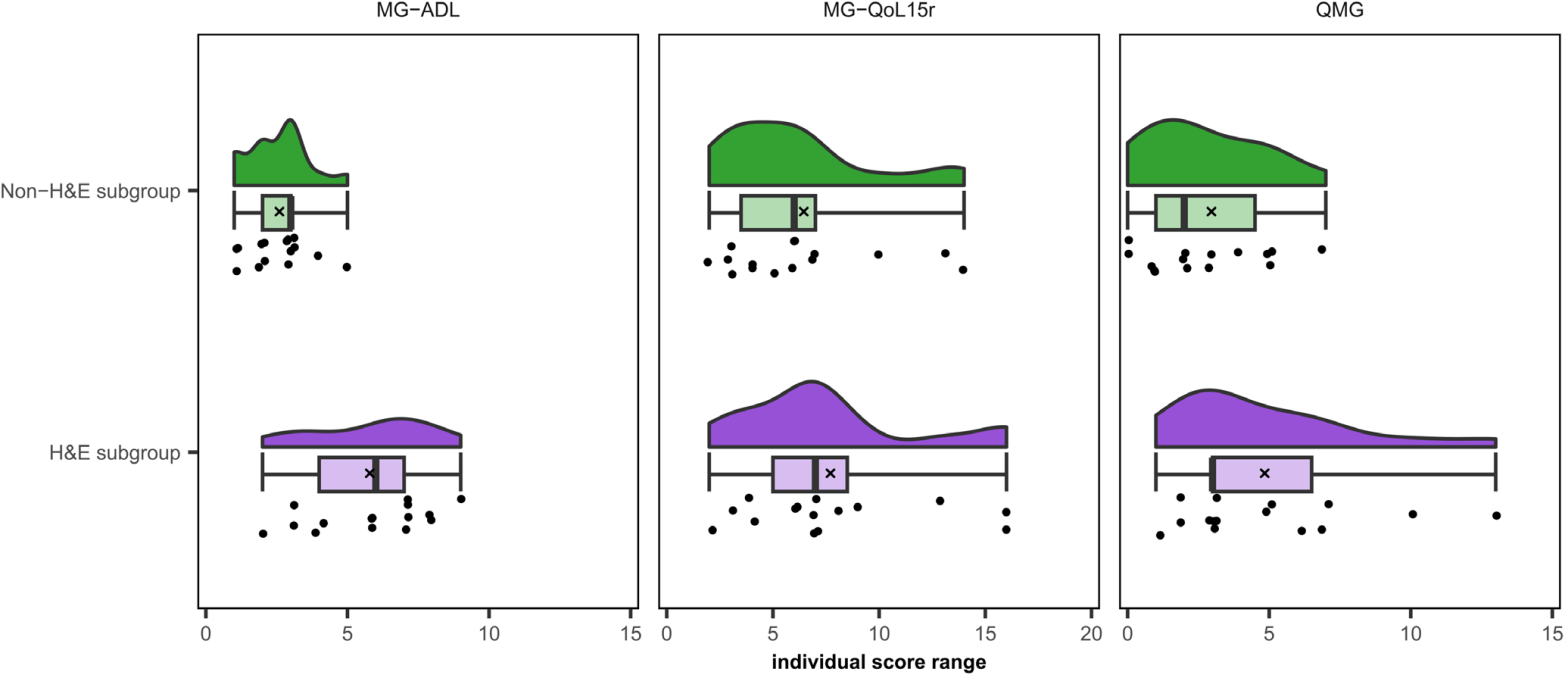
Ranges of clinical score variation throughout the study in the intervention subgroups. The black dots represent the range in clinical score variation per patient in the H&E (Hospitalization & Exacerbation) and Non‐H&E (Non‐Hospitalization & Exacerbation) subgroup, defined as the difference between maximum and minimum clinical score value (H&E subgroup includes patients with MG‐related hospitalization and/or exacerbation during the study; Non‐H&E subgroup includes patients without such deterioration). The mean range per subgroup is marked by a cross. MG‐ADL, Myasthenia gravis activities of daily living; MG‐QoL15r, Myasthenia gravis Quality of life, revised version; QMG, quantitative Myasthenia gravis score.

In the H&E subgroup, patients showed a greater range in clinical score variation throughout the study (MG‐ADL, MG‐QoL15r, and QMG) compared to those in the Non‐H&E subgroup, defined as the difference between maximum and minimum clinical score value. The most pronounced difference between both subgroups was observed in MG‐ADL (mean range: 5.8 vs. 2.6 points) (Figure 3). All 15 patients (100%) in the H&E subgroup had ≥ 2 points range of variation in MG‐ADL versus 12 patients (80%) in the Non‐H&E subgroup. Regarding QMG scores, 12 patients (80.0%) in the H&E subgroup had a ≥ 3 points range of variation versus only seven patients (46.7%) in the Non‐H&E subgroup (Table S3). This represents a purely descriptive observation without any statistical significance.

**FIGURE 4:**
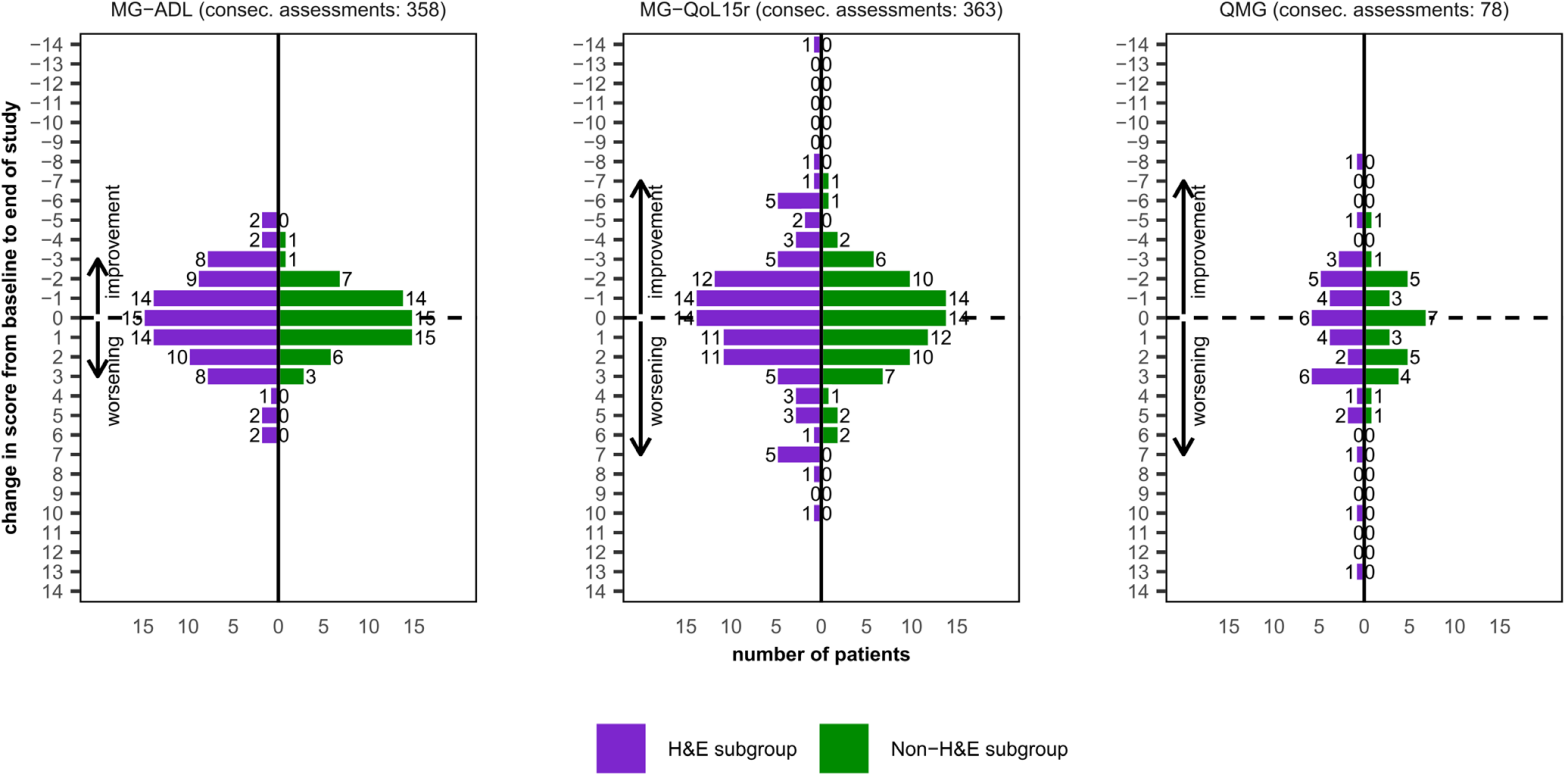
Changes in clinical scores between two consecutive assessments in intervention subgroups. The figure displays the number of patients in the H&E (Hospitalization & Exacerbation) and Non‐H&E (Non‐Hospitalization & Exacerbation) subgroup who exhibited specific changes in clinical scores at least once between two consecutive assessments during the study (H&E subgroup includes patients with MG‐related hospitalization and/or exacerbation during the study; Non‐H&E subgroup includes patients without such deterioration). MG‐ADL, Myasthenia gravis activities of daily living; MG‐QoL15r, Myasthenia gravis quality of life, revised version; QMG, quantitative Myasthenia gravis score.

Across both the H&E and Non‐H&E subgroups, changes in MG‐ADL, MG‐QoL15r, and QMG scores between two consecutive assessments were generally small. However, patients of the H&E subgroup showed more pronounced changes in clinical scores between two consecutive assessments, particularly in MG‐ADL, compared to the Non‐H&E subgroup; for example, ten patients (66.6%) in the H&E subgroup had at least once a 2‐point increase in MG‐ADL, compared to six patients (40.0%) in the Non‐H&E subgroup. Similarly, six (40.0%) patients in the H&E subgroup had at least once a 3‐point increase in QMG, compared to four patients (26.6%) in the Non‐H&E subgroup (Figure 4).

For PASS evaluation, a higher proportion of patients in the H&E subgroup compared to the Non‐H&E subgroup (*n* = 7 vs. *n* = 2) showed alternating responses between “Yes” and “No” when asked the question: “If you think about how the myasthenia has affected you overall over the last few weeks and you imagine that this condition will continue for the next few months, would that be acceptable to you?” (Figure S4).

### 3.6 Communication and Intervention Behavior in the H&E and Non‐H&E Subgroups

**FIGURE 5:**
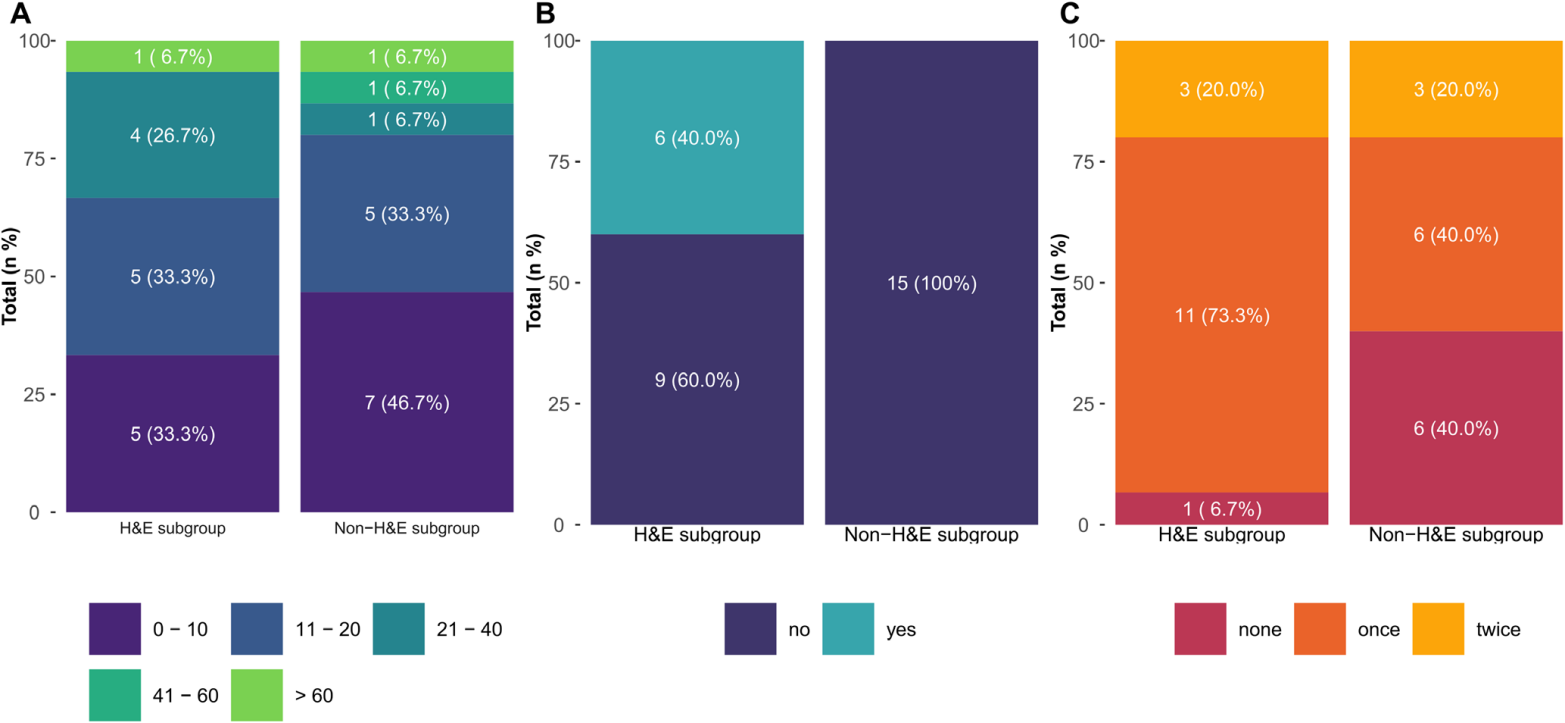
Communication and intervention behavior in intervention subgroups. Absolute and relative frequencies for H&E (Hospitalization & Exacerbation) subgroup and Non‐H&E (Non‐Hospitalization & Exacerbation) subgroup (H&E subgroup includes patients with MG‐related hospitalization and/or exacerbation during the study; Non‐H&E subgroup includes patients without such deterioration) f.l.t.r. for number of messages sent by patients via the chat during the study, change of steroid dosage between baseline visit and end of study visit, and frequency of interventions, defined as a physician‐initiated contact with the patient via the chat following the review of monitoring parameters during the two scheduled telemedical check‐ups (TCUs).

Within the intervention group, which received additional telemedical support including a chat function for physician contact, communication and intervention behavior differed between the H&E and Non‐H&E subgroups. Messaging activity was higher in the H&E subgroup, with 60.0% of patients sending between 11 and 40 messages over the study period, compared to 40.0% in the Non‐H&E subgroup (Figure 5). In contrast, 33.3% of patients in the H&E subgroup sent ≤ 10 messages, compared to 46.7% in the Non‐H&E subgroup. Regarding steroid use, more patients in the H&E subgroup had their steroid dosage adjusted (40.0%) by the physician, with all changes directly communicated to the patient through the MyaLink chat. In contrast, no steroid dosage changes were made in the Non‐H&E subgroup. Most patients in the H&E subgroup (93.3%) received at least one intervention during a TCU, defined as a physician‐initiated contact through the chat following the review of monitoring parameters (e.g., change in medication, further consultation based on deterioration of monitoring data), whereas only 60.0% of the Non‐H&E subgroup received at least one intervention.

Among all patients in the intervention group, 20 patients (66.7%) experienced at least once a ≥ 2‐point increase in MG‐ADL score between two consecutive assessments. Of these, 11 patients (55.0%) proactively contacted the physician via the chat within the ±7‐day window surrounding the day of MG‐ADL score increase (“high increase period”). These interactions were only related to MG‐specific concerns such as MG treatment adjustments and/or myasthenic complaints. During these high increase periods, patients sent an average of 0.7 MG‐related messages, compared to only 0.4 messages during a stable 14‐day period without an MG‐ADL increase ≥ 2‐point (Table 2).

**TABLE 2:** MG‐related communication behavior for patients in the intervention group with at least one MG‐ADL increase ≥ 2 points between two consecutive measurements.

	Patients with at least one MG‐ADL ≥ 2 points increase between two consecutive assessments *N* = 20	
	High increase period^a^	Stable period^b^
Period duration in days, mean	28.7	56.3
Patients with proactive MG‐related messages^c^, *n* (%)	11 (55.0)	13 (65.0)
Proactive MG‐related messages from patients^c^, *n*	26	27
Per period, mean	1.3	1.4
Per 14 days within period, mean	0.7	0.4

Abbreviations: MG, Myasthenia gravis; MG‐ADL, Myasthenia gravis activities of daily living.

^a^ Defined by including days within a ±7‐day window around day of MG‐ADL score increase of ≥ 2 points. In cases of multiple increases ≥ 2 points in MG‐ADL within a single patient, overlapping ±7‐day windows were merged, and overlapping days were counted only once for calculating the high increase period.

^b^ Defined as the individual study period in days minus the individual high increase period in days.

^c^ MG‐related messages from patients only included inquiries about MG treatment and/or myasthenic complaints.

## 4 Discussion

This analysis of the MyaLink study data evaluated changes in clinical outcomes in the patients who were allocated to the MyaLink remote monitoring group (intervention group) compared to the standard care control group. We found considerable variation in clinical outcome measures through the 12‐week study, without significant differences between groups. The intervention group had fewer exacerbations but more MG‐related hospitalizations, but this was not statistically significant. The difference might be partially explained by the fact that patients in the intervention group were more severely affected according to MGFA status classification at baseline. However, it is also possible that at least to some extent this is due to fluctuations related to the disease due to the limited sample size. If this is indeed the nature of the disease, our data highlight the potential of remote monitoring to detect individual MG‐related fluctuations reflecting disease status that may be missed in traditional care settings. Our study demonstrates that MyaLink is a powerful tool for capturing the highly heterogeneous disease course.

By leveraging fine‐grained data and digital assessments of both subjective (PROMs) and objective data (external devices), this offers a unique window into the individual disease course between clinical visits, effectively “unveiling the blackbox” between in‐person appointments. This highlights the potential for early symptom detection and interventions as necessary that could positively impact the clinical course.

Interestingly, patients underwent spirometry measurements much more frequently than required by protocol, suggesting that they might have found this a reassuring self‐evaluation of respiratory status. When comparing H&E and Non‐H&E subgroups, the H&E subgroup showed greater mean range of variation in overall clinical scores throughout the study as well as greater fluctuations in clinical scores between two consecutive assessments, particularly for MG‐ADL. While these observations are purely exploratory and hypothesis‐generating and require confirmation in larger, prospective cohorts, they may point toward the potential of granular longitudinal data to identify patients with more unstable or “brittle” disease courses. First complementary analyses investigating potential predictive digital markers of exacerbation have already been undertaken in a companion paper from our study applying machine learning approaches to wearable and patient‐reported data [29].

If validated in future research, such variability patterns might support a more refined clinical differentiation of disease control between visits. In the future, a better understanding of individual disease course may help for identification of parameters as potential early indicators of clinical worsening (i.e., “digital biomarkers”), which have been described in other autoimmune neurological disorders [30] like multiple sclerosis [31, 32] and other chronic disorders [33]. This also requires more research to identify factors which shape patients' reporting practices and how these relate to objectively observed changes in monitoring parameters. In MG, this could guide treatment decisions and allow timely interventions, ultimately leading to more effective management of MG. The higher number of messages sent by patients in the H&E subgroup, difference in changes in steroid dosage between these groups and more frequent interventions at TCUs (physician‐initiated contact via the chat following the review of monitoring parameters), suggests that this subgroup may have had a greater need for medical care and support. While the value of digitally‐assessed PROMs via a smartphone particularly for symptom characterization during MG exacerbation was also shown in one other study [34], overall, comprehensive monitoring studies including wearable devices still remain underexplored in MG. While some studies have investigated the utility of specific/single telemedical tools or scales for symptom assessment in MG [35, 36, 37, 38, 39, 40, 41], including the use of machine‐learning algorithms for remote symptom evaluation [42, 43, 44], most of these studies have focused on the assessment of ocular and facial weakness. Only few studies in other rare neuromuscular disorders have digitally assessed clinical outcomes using an app/software, but none enabling direct patient‐physician interaction. Most of these studies examined amyotrophic lateral sclerosis [45, 46, 47, 48], and one each in facioscapulohumeral muscular dystrophy [49], Friedreich's ataxia (but only recorded for one week) [50], GM2 gangliosidosis [51] and one study examining multiple rare neuromuscular disorders [52].

Despite these promising aspects of remote monitoring, one of the key issues for successful implementation in clinical care will be the efficient usage and integration of such data into clinical decision‐making processes. A careful assessment of data quality and interpretation within the clinical context and a thorough consideration of the right monitoring intensity for different patient groups is necessary (e.g., those with a short disease duration, highly active disease and/or fast‐acting treatment regimens may require more frequent assessments).

The study limitations include a small sample size and a short observation period. The study was conducted in a single academic center, which may limit the generalizability of the results. The cohort also showed a female predominance, which is characteristic of early‐onset MG but may be somewhat overrepresented in this study compared with population‐based cohorts, particularly with regard to late‐onset MG, where male predominance is typically observed. Patients also had to have some digital literacy; that could be a barrier to integration in clinical practice. Furthermore, the current lack of reimbursement possibilities is a limiting factor for successful implementation in general practice.

In conclusion, our study provides promising evidence for the potential of remote monitoring and telemedical treatment provided by solutions such as MyaLink to capture the heterogeneous course of the disease. These unprecedented real‐time insights that are typically missed in standard care settings could complement and enhance current MG care. Especially high‐risk patients could benefit from early detection of symptom deterioration and more accessible medical care. More extensive research is needed to understand the impact of telemedical care on long‐term clinical outcomes and to optimize its integration into routine clinical practice.

## Author Contributions

Conceptualization: M.S., S.L., and L.G. Methodology: M.S., S.L., L.G., and M.M. Investigation: M.S. and S.L. Data curation: M.S. and M.M. Writing – original draft: M.S. and M.M. Writing – review and editing: M.M., M.H., F.S., S.H., H.P., P.M., C.D., P.D., H.S., P.N., A.M., L.G., and S.L. Formal Analysis: M.M. Project administration: M.S. and S.L. Funding acquisition: M.S. and A.M. Resources: A.M.

## Funding

The project was funded by the Deutsche Forschungsgemeinschaft (DFG, German Research Foundation) (553539684). Argenx, UCB and Hormosan provided a grant to support the study but were not involved in the design of the study. The project was partly funded and supported by the Berlin Institute for Health/Charité Universitätsmedizin Berlin.

## Conflicts of Interest

M.S. has received speaker's honoraria and honoraria for attendance at advisory boards from Argenx and Alexion. M.H. has received speaker's honoraria from Argenx and speaker's honoraria and honoraria for attendance at advisory boards from Alexion. F.S. received travel/accommodation/meeting expenses from Alexion Pharmaceuticals and argnx and received speaking honoria and honoria for attendance at advisory boards from Alexion Pharmaceuticals, argnx and UCB pharma. She receives financial research support (paid to her institution) from Alexion Pharmaceuticals. She serves as a member of the medical advisory board of the German Myasthenia Gravis Society e.V. S.H. has received speaker honoraria from Alexion, argenx BV, Grifols, Johnson&Johnson, Merck, Roche, UCB; honoraria for attendance at advisory boards from Alexion, argenx BV, Johnson&Johnson, Novartis and Roche, research grants from argenx BV and Johnson&Johnson and serves as a member of the medical advisory board of the German Myasthenia Gravis Society. P.M. received travel/accommodation/meeting expenses from UCB pharma. P.N. received grant support from PCORI, Alexion, Dianthus, and Janssen. P.N. serves as consultant/advisory board member for Alexion, Amgen, Argenx, CVS Caremark, Dianthus, GSK, Immune Abs, Janssen, Novartis, and UCB. P.N. serves as DSMB for Argenx, Sanofi. Royalties: Springer Nature. M.H. has received speaker's honoraria from Argenx and speaker's honoraria and honoraria for attendance at advisory boards from Alexion. A.M. received speaker's honoraria, served as an advisory board/DSMB member and consultant, and has received research grants (paid to his institution) from Alexion AstraZeneca Rare Disease, Amgen, argenx, Axunio, Desitin, Genpharm, Grifols, Hormosan, Immunovant, Janssen, Merck, Novartis, Octapharma, Regeneron, Sanofi and UCB. He is chairman of the *Association for Research of Myasthenic Syndromes in Germany* and member of the medical advisory board of the German Myasthenia Gravis Society. L.G. has received speaker's honoraria from Alexion and Roche. S.L. received travel/accommodation/meeting expenses from Alexion Astra Zeneca Rare Disease, Argenx, Johnsson&Johnsson, and UCB received speaking honoria and honoria for attendance at advisory boards from Alexion Astra Zeneca Rare Disease, Argenx, Biogen, Hormosan, Huma, Johnsson&Johnsson, Merck, Roche, StreamedUp and UCB. She received financial research support (paid to her institution) from Ad Scientiam, Alexion Pharmaceuticals, Argenx, Hormosan and UCB. S.L. is a shareholder of mamahealth GmbH. M.S., S.L. and L.G. are co‐founders and shareholders of RareLink digital health GmbH. All other authors have no conflicts of interest to report.

## Supporting information


**Data S1:** acn370293‐sup‐0001‐TableS1‐S3‐FigureS1‐S4.docx.

## Data Availability

The data that support the findings of the study are available from the corresponding author upon reasonable request.
