# Supplementary material for: Remote Monitoring in Myasthenia Gravis: Exploring Symptom Variability
Source: Ann Clin Transl Neurol. 2025 Dec 21;13(6):1106–17. doi: 10.1002/acn3.70293 (PMC13251423; doi:10.1002/acn3.70293)
Supplement: Supplementary file 1 — Data S1: acn370293‐sup‐0001‐TableS1‐S3‐FigureS1‐S4.docx. [file ACN3-13-1106-s001.docx]

**Supplemental Materials**

**Table S1: Clinical outcome parameters at baseline, end of the study, and score changes between both visits for the intervention and control group**

|  | **Baseline** | | **End of study** | | **Median of differences (EoS – BL)** | |
| --- | --- | --- | --- | --- | --- | --- |
|  | Intervention group  n=30 | Control group  n=15 | Intervention group  n=29^1^ | Control group  n=15 | Intervention group  n=29^1^ | Control group  n=15 |
| **MG-ADL** |  |  |  |  |  |  |
| Median [IQR; min-max] | 10.0 [5.3, 11.0; 1-14] | 7.0 [5.5, 9.5; 4-14] | 0.0 [-1.0, 2.0; -3-6] | 0.0 [-1.5, 1.5; -4-4] | 10.0 [7.0, 12.0; 1-17] | 6.0 [5.5, 10.5; 2-14] |
| **MG-QoL15r** |  |  |  |  |  |  |
| Median [IQR; min-max] | 15.5 [12.3, 20.8; 4-28] | 16.0 [11.5, 22.0; 10-24] | 0.0 [-2.3, 2.0; -12-14] | 1.0 [-1.0, 2.0; -12-13] | 18.0 [14.0, 20.0; 1-26] | 13.0 [10.0, 22.5; 8-27] |
| **QMG** |  |  |  |  |  |  |
| Median [IQR; min-max] | 15.0 [12.3, 17.0; 1-25] | 16.0 [14.5, 16.5; 11-25] | 1.0 [0.0, 3.0; -5-13] | -1.0 [-2.0, 2.5; -4-6] | 15.0 [13.0, 20.0; 1-30] | 17.0 [14.0, 19.5; 9-24] |
| **CFS** |  |  |  |  |  |  |
| Median [IQR; min-max] | 20.0 [16.3, 23.0; 9-29] | 20.0 [18.0, 22.5; 11-30] | -1.0 [-3.0, 2.0; -5-5] | -4.0 [-6.5, 3.5; -10-12] | 20.0 [16.0, 22.0; 5-27] | 18.0 [15.5, 21.5: 7-33] |
| **HADS** |  |  |  |  |  |  |
| Median [IQR; min-max] | 12.0 [6.0, 16.0; 2-26] | 18.0 [11.0, 22.5; 2-26] | -1.0 [-2.0, 2.0; -10-8] | -1.0 [-2.0, 2.5; -14-21] | 14.0 [4.0, 16.0; 0-28] | 18.0 [10.0, 22.5; 0-28] |
| **SSQ** |  |  |  |  |  |  |
| Median [IQR; min-max] | 50.0 [35.0, 70.0; 25-90] | 55.0 [40.0, 65.0; 15-80] | 0.0 [-12.0, 0.0; -50-20] | 0.0 [-20.0, 9.5; -35-45] | 45.0 [30.0, 65.0; 18-100] | 50.0 [27.5, 62.5; 10-90] |
| **FVC, in litres** |  |  |  |  |  |  |
| Median [IQR; min-max] | 3.4 [2.7, 3.7; 2.1-4.8] | 3.3 [2.7, 3.8; 2.0-6.7] | -0.2 [-0.4, 0.2; -2.3-2.6] | 0.0 [-0.2, 0.3; -1.0-1.8] | 3.2 [2.5, 3.7; 0.6-6.9] | 3.5 [3.0, 3.9;2.5-6.4] |
| **SBCT** |  |  |  |  |  |  |
| Median [IQR; min-max] | 19.5 [12.3, 22.8; 7-34] | 20.0 [15.0, 24.5; 7-30] | 2.0 [-1.0, 8.0; -4-12] | 1.0 [-4.5, 5.0; -9-11] | 21.0 [15.0, 30.0; 7-40] | 21.0 [12.5, 25.5; 11-28] |
| **EQ5D5L-Index** |  |  |  |  |  |  |
| Median [IQR; min-max] | 0.7 [0.5, 0.8; 0.1-1.0] | 0.7 [0.6, 0.7; 0.3-0.9] | -0.1 [-0.1, 0.0; -0.4-0.3] | 0.0 [-0.1, 0.1; -0.8-0.6] | 0.6 [0.3, 0.7; 0.0-1.0] | 0.7 [0.6, 0.8; -0.1-0.9] |

^1^ one patient had incomplete follow-up (drop out)
Raw data for clinical outcome parameters at baseline and end of study visit, and the median of individual score changes between baseline and end of study visit per outcome and per group are presented. BL – Baseline; EoS - End of study; MG-ADL – Myasthenia gravis Activities of daily living; MG-QoL15r - Myasthenia gravis Quality of life, revised version; QMG - Quantitative Myasthenia gravis score; CFS - Chalder Fatigue Scale; HADS - Hospital Anxiety and Depression Scale; SSQ - Single Simple Question; FVC - Forced vital capacity; SBCT - Single Breath Count Test; EQ5D5L - health-related quality of life (developed by the EuroQol Group)

**
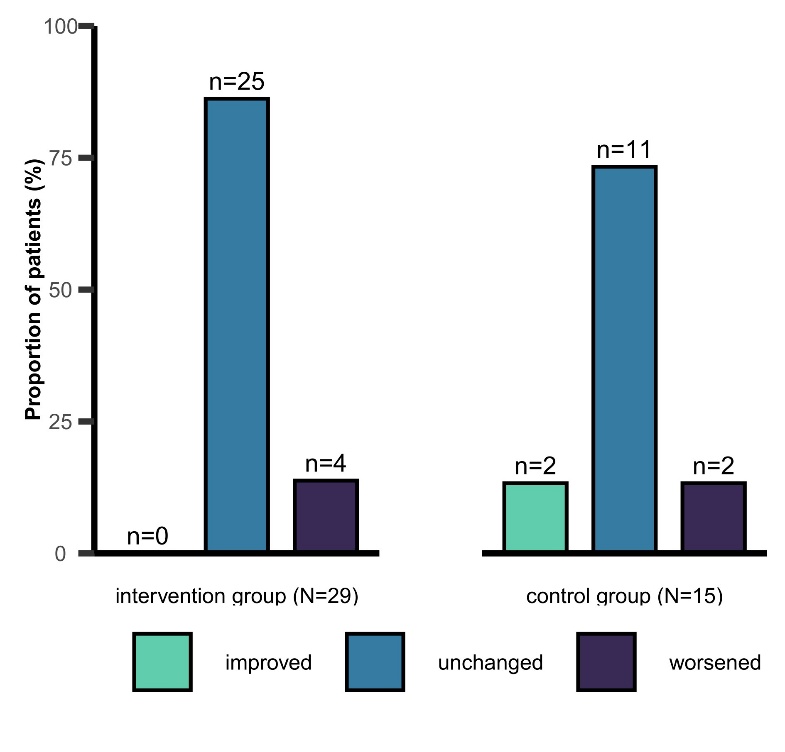
**

**Figure S1: MGFA-PIS at the end of the study**
MGFA-PIS assessment at the end of the study per category in the intervention group and control group. MGFA-PIS was unavailable for one dropout in the intervention group. MGFA-PIS - MGFA Post-intervention status.


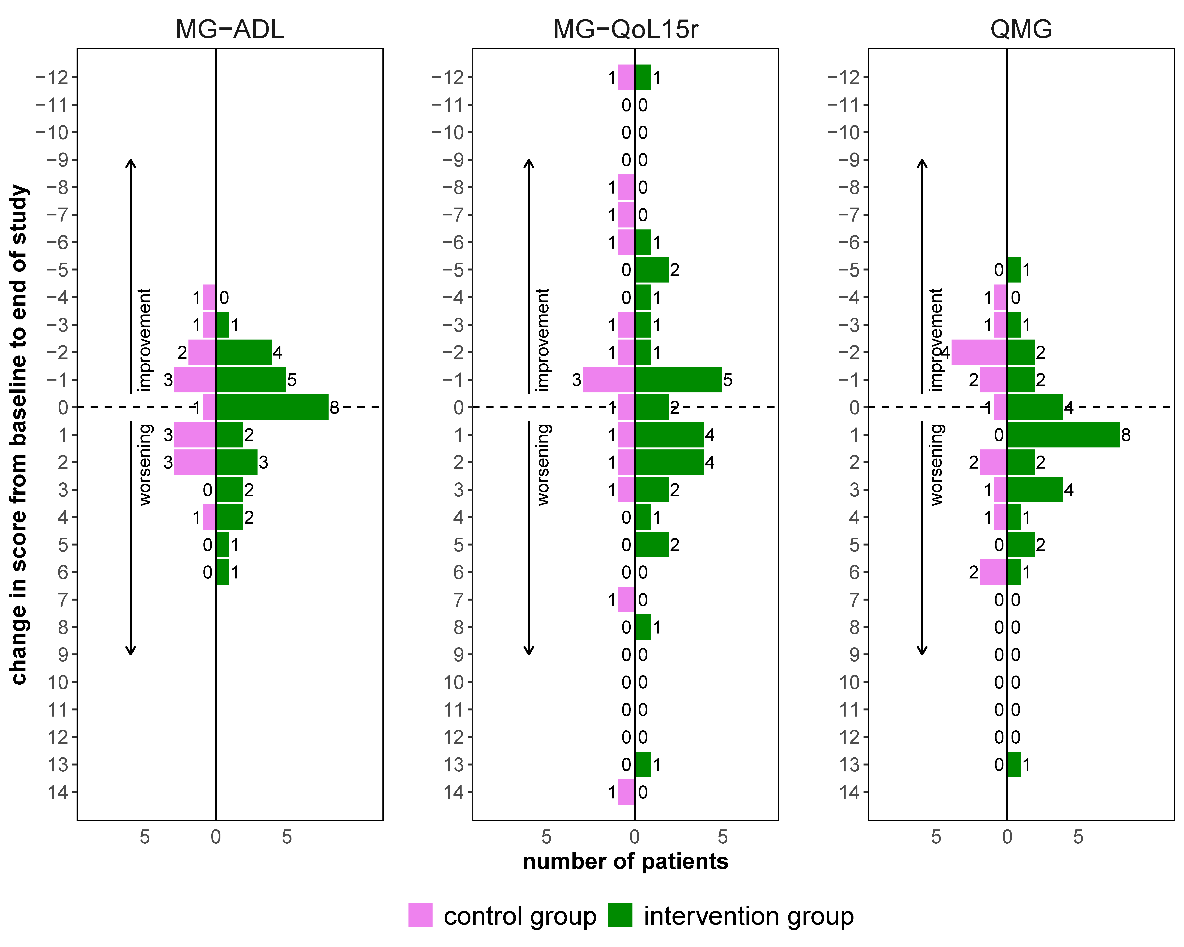


**Figure S2: Individual change in clinical scores from baseline to end of study in the intervention and control group**

Individual change in clinical scores from baseline to end of study in the intervention group (n=29, one drop out) and control group (n=15). MG-ADL - Myasthenia gravis Activities of daily living; MG-QoL15r - Myasthenia gravis Quality of life, revised version; QMG - Quantitative Myasthenia gravis score.

**Table S2**: **MG-related hospitalizations and exacerbations**

|  | Overall n=44^1^ | Intervention group  n=29^1^ | Control group  n=15 | Odds Ratio | 95% CI |
| --- | --- | --- | --- | --- | --- |
| Patients with self-reported exacerbation(s) and/or MG-related hospitalization(s), n (%) | 23 (52.3) | 15 (51.7) | 8 (53.3) | 0.9 | 0.3-3.3 |
| Patients with self-reported exacerbation(s), n (%) | 21 (47.7) | 13 (44.8) | 8 (53.3) | 0.7 | 0.2-2.5 |
| Patients with MG-related hospitalization(s), n (%) | 6 (13.6) | 5 (17.2) | 1 (6.7) | 2.9 | 0.3-27.6 |
| Number of MG-related hospitalizations^2^ | 9 | 8 | 1 |  |  |
| MGFA status classification  (at baseline)^3^, n (%) |  |  |  |  |  |
| IIIb | 2 (33.3) | 2 (40.0) | 0 (0.0) |  |  |
| IVb | 4 (66.7) | 3 (60.0) | 1 (100) |  |  |
| Hospitalization duration |  |  |  |  |  |
| 1-5 days | 2 (22.2) | 2 (25.0) | 0 (0.0) |  |  |
| 6-14 days | 1 (11.1) | 1 (12.5) | 0 (0.0) |  |  |
| 15-30 days | 5 (55.6) | 4 (50.0) | 1 (100) |  |  |
| > 30 days | 1 (11.1) | 1 (12.5) | 0 (0.0) |  |  |
| Type of ward (multiple answers possible) |  |  |  |  |  |
| Emergency room | 1 (11.1) | 1 (12.5) | 0 (0.0) |  |  |
| General ward | 8 (88.9) | 7 (87.5) | 1 (100) |  |  |
| Intensive care unit | 2 (22.2) | 1 (12.5) | 1 (100) |  |  |
| Type of therapy (multiple answers possible) |  |  |  |  |  |
| IVIg | 5 (55.6) | 4 (50.0) | 1 (100) |  |  |
| Plasmapheresis/immunoadsorption | 2 (22.2) | 1 (12.5) | 1 (100) |  |  |
| Supportive therapy^4^  (multiple answers possible) |  |  |  |  |  |
| Non-invasive ventilation (NIV) | 2 (22.2) | 1 (12.5) | 1 (100) |  |  |

^1^ one patient had incomplete follow-up (drop out)
^2^ multiple hospitalizations per patient possible ^3^ n (%) among hospitalized patients
^4^ excluding two patients, one in each group, with only clinical observation
MG – Myasthenia gravis; MGFA – Myasthenia gravis Foundation of America; IVIg - intravenous immunoglobulin; CI – confidence interval.


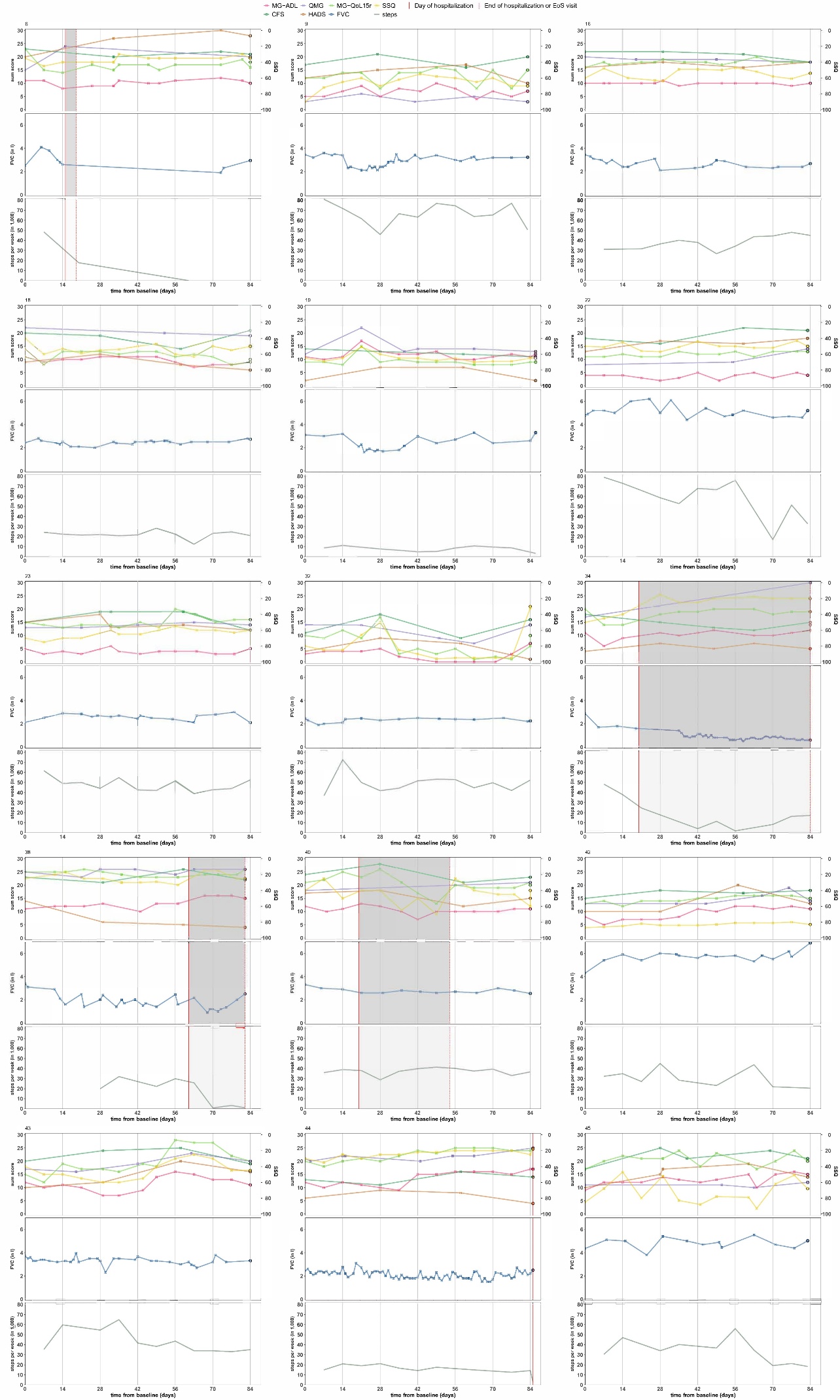


**Figure S3: Individual longitudinal changes of different monitoring parameters in the H&E subgroup**

The time course of the monitoring parameters is illustrated in individual diagrams for each of the 15 patients in the H&E subgroup (H&E subgroup includes patients with MG-related hospitalization and/or exacerbation during the study). From top to bottom, the plot shows patient-related outcome measures (PROMs), forced vital capacity (FVC) and weekly step count. Each measurement is represented by a box, and the final recorded value for each patient is highlighted with a circle. Periods of hospitalization are indicated by red horizontal lines. MG-ADL – Myasthenia gravis Activities of daily living, MG-QoL15r - Myasthenia gravis Quality of life, revised version, QMG - Quantitative Myasthenia gravis score, CFS - Chalder Fatigue Scale, HADS - Hospital Anxiety and Depression Scale, FVC - Forced vital capacity, SBCT - Single Breath Count Test.

**Table S3: Range of variation in clinical score across all measurements per patient in the intervention subgroups**

|  | **Patients with score**  **range ≥ 2 points** | | **Patients with score**  **range ≥ 3 points** | | **Patient with score**  **range ≥ 5 points** | |
| --- | --- | --- | --- | --- | --- | --- |
| Clinical score | H&E subgroup | Non-H&E subgroup | H&E subgroup | Non-H&E subgroup | H&E subgroup | Non-H&E subgroup |
| MG-ADL, n (%) | 15 (100) | 12 (80.0) | 14 (93.3) | 8 (53.3) |  |  |
| MG-QoL15r, n (%) |  |  | 14 (93.3) | 14 (93.3) | 11 (73.3) | 9 (60.0) |
| QMG, n (%) |  |  | 12 (80.0) | 7 (46.7) | 7 (46.7) | 4 (26.7) |

Patients in the intervention subgroups (H&E subgroup includes patients with MG-related hospitalization and/or exacerbation during the study; Non-H&E subgroup includes patients without such deterioration) who had a specific individual score range (maximum-minimum score) across all individual measurements for MG-ADL, MG-QoL15r, and QMG. Score changes could occur in either direction (increase or decrease). MG-ADL – Myasthenia gravis Activities of daily living; MG-QoL15r – Myasthenia gravis Quality of life, revised version; QMG - Quantitative Myasthenia gravis score.

**
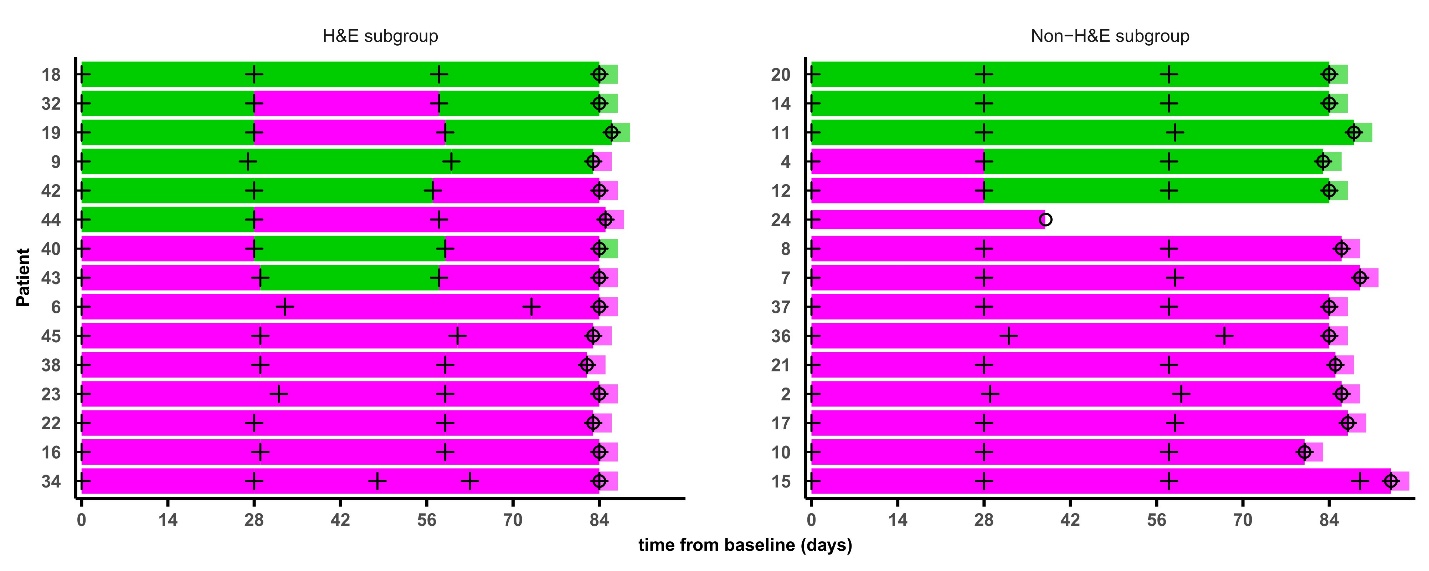
**

**Figure S4: PASS assessments in intervention subgroups**Individual responses in repeated PASS (Patient Acceptable Symptom State) assessments in the intervention subgroups (H&E subgroup includes patients with MG-related hospitalization and/or exacerbation during the study; Non-H&E subgroup includes patients without such deterioration). Each PASS assessment timepoint is indicated as “+”, intervals between PASS assessments are visualized as bars (red bar indicating previous PASS answer was “no”, green bar indicating previous PASS answer was “yes”). The end of study visit is indicated as “o”.
